# Medical College Notes

**Published:** 1891-07

**Authors:** 


### Medical College Notes.

THE Kentucky School of Medicine held its commencement exercises at Macauley's Theater, Louisville, on Thursday, June 18, 1891, at which 155 new doctors were commissioned. The success of this school, under the management of Dr. W. H. Wathen as Dean, has been almost phenomenal, 400 matriculates having registered during the college year just closed.

---

THE following changes in the Medical Department of Niagara University are announced: Dr. Frank H. Potter has resigned as Lecturer on Diseases of the Nose and Throat, in which department he has served most acceptably, to take a position in Buffalo Medical College. Dr. W. Scott Renner, of this city, has been appointed to succeed him. Dr. Walter D. Green, health physician of this city, has been called to succeed Dr. F. A. Harrington in the Department of Hygiene. Dr. Harry A. Wood, also of Buffalo, has been elected Lecturer on Materia Medica, to coöperate with Prof. Persons and with Dr. Henry C. Buswell, who has been elected Adjunct Professor of Materia Medica and Therapeutics. Dr. L. Bradley Dorr has been elected Adjunct Professor of General Chemistry and Physics, and Dr. William C. Krauss, Professor of Pathology. The chair of Surgery has been divided; Dr. Herman Mynter takes Operative Surgery and Clinical Surgery, and Dr. Herbert Mickle has been transferred from the chair of Anatomy to that of Professor of Principles and Practice of Surgery. Dr. Eugene A. Smith has been elected Professor of General and Descriptive Anatomy, and Dr. John D. Flagg, Professor of Surgical and Practical Anatomy. Frederick H. Mills, who has been engaged in special study at Princeton University for some time, has been appointed Demonstrator of Chemistry; Dr. Jacob S. Peterson, Clinical Assistant in Obstetrics; and Dr. Matthew J O'Connell, Clinical Assistant in Insanity. The next course of lectures has been considerably prolonged, with the intention of omitting the Spring course. The lectures will begin Sept. 21, 1891, and close May 3, 1892. A valuable addition to the already excellent clinical facilities of the school is the Lying-in Asylum on Edward street. The obstetric privileges of senior students are thus very largely increased. This school is reported to be seriously considering the further extension of its course to a compulsory attendance upon four annual sessions, which, if adopted, will keep it still in the advance line. This, we are informed, will not be done immediately, though we think it ought to be.

